# The predicted effect of changes in cervical screening practice in the UK: results from a modelling study

**DOI:** 10.1038/sj.bjc.6602002

**Published:** 2004-07-13

**Authors:** K Canfell, R Barnabas, J Patnick, V Beral

**Affiliations:** 1 Cancer Research UK Epidemiology Unit, University of Oxford, Gibson Building, Radcliffe Infirmary, OX2 6HE, UK; 2 Director, NHS Cancer Screening Programmes, The Manor House, 260 Ecclesall Road South, Sheffield, S11 9PS, UK

**Keywords:** cervix neoplasms, mass screening, models, biological

## Abstract

In 2003, the National Health Service Cervical Screening Programme (NHSCSP) announced that its screening interval would be reduced to 3 years in women aged 25–49 and fixed at 5 years in those aged 50–64, and that women under 25 years will no longer be invited for screening. In order to assess these and possible further changes to cervical screening practice in the UK, we constructed a mathematical model of cervical HPV infection, cervical intraepithelial neoplasia and invasive cervical cancer, and of UK age-specific screening coverage rates, screening intervals and treatment efficacy. The predicted cumulative lifetime incidence of invasive cervical cancer in the UK is 1.70% in the absence of screening and 0.77% with pre-2003 screening practice. A reduction in lifetime incidence to 0.63% is predicted following the implementation of the 2003 NHSCSP recommendations, which represents a 63% reduction compared to incidence rates in the UK population if it were unscreened. The model suggests that, after the implementation of the 2003 recommendations, increasing the sensitivity of the screening test regime from its current average value of 56 to 90% would further reduce the cumulative lifetime incidence of invasive cervical cancer to 0.46%. Alternatively, extending screening to women aged 65–79 years would further reduce the lifetime incidence to 0.56%. Screening women aged 20–25 years would have minimal impact, with the cumulative lifetime incidence decreasing from 0.63 to 0.61%. In conclusion, the study supports the 2003 recommendations for changes to cervical screening intervals.

The implementation of the National Health Service Cervical Screening Programme (NHSCSP) in 1988 resulted in a substantial reduction in the overall incidence of cervical cancer (Quinn *et al*, 1999). The NHSCSP has invited women aged 20–64 years for cervical cancer screening every 3–5 years, depending upon the specific policy of the local Health Authority. In 2003, the NHS announced that women aged 25–49 years will be invited for screening every 3 years, while those aged 50–64 should be invited every 5 years. It was also recommended that women under 25 years should no longer be screened. The new screening interval recommendations are based on the estimated duration of the protective effect of screening in different age groups and are the result of an audit of the screening histories of women with frankly invasive cancer and age-matched controls (Sasieni *et al*, 2003).

In order to investigate the likely impact of changes in screening practice upon the incidence of invasive cervical cancer in the UK, we developed a mathematical model of cervical human papillomavirus (HPV) infection, cervical intraepithelial neoplasia (CIN) and invasive cervical cancer, and of screening in the UK population. The model was developed so that parameters such as the frequency of screening within various age groups and sensitivity of the screening test could be altered in a systematic way. We then evaluated the impact of the 2003 recommendations on cervical cancer incidence rates in the UK and also explored the impact of possible further changes in screening practice.

## MATERIALS AND METHODS

### Model description

**Figure 1:**
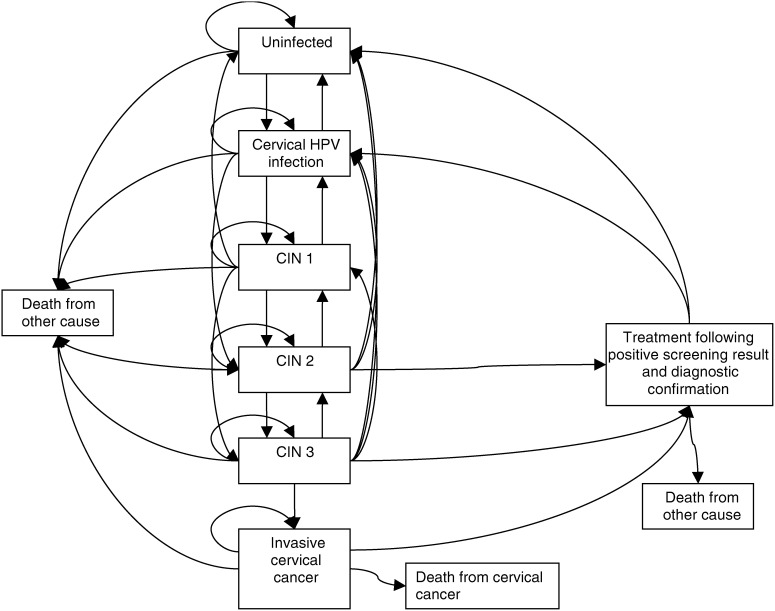
Markov model incorporating states for cervical HPV infection, CIN and treatment of screen-detected disease.

A Markov simulation was developed with two components. The first component involved the development of a mathematical model of HPV natural history, progression to CIN and cervical cancer within the UK population. The second model component dealt with the screening and treatment of preneoplastic disease. The health states in the final model are illustrated in Figure 1

. The simulation follows a cohort of women, year by year, from age 16 to 80 years. The model assumes that the women are always in one of nine possible states, with transitions between states occurring annually according to age-dependent probabilities.

### Natural history model component

The simulation commences with a cohort of 100 000 16-year-old girls and it is assumed that the majority (approximately 89%) of these are HPV naïve, approximately 10% have asymptomatic cervical HPV infection and 1% have CIN 1. Each year, an age-specific risk of acquiring an HPV infection is applied to women in the cohort. The state of ‘cervical HPV infection’ in Figure 1 represents the presence of PCR-detectable HPV DNA without the development of CIN. The next four states in Figure 1 represent further stages of disease pathogenesis – CIN 1, CIN 2, CIN 3 and invasive cervical cancer, which includes both microinvasive (Stage 1A) and frankly invasive (Stages 1B+) cancer. Women may die from cervical cancer (death from cervical cancer) and women in each of the other states are subject to age-specific mortality from other causes (death from other cause). Age-specific death rates for causes other than cervical cancer were obtained using overall mortality rates for England and Wales and subtracting the mortality rates for cervical cancer (Office of National Statistics, 2003a). At each stage of infection, HPV infection or CIN lesions can regress, persist or progress.

The parameters used for HPV and CIN regression and progression were based on a systematic review of the relevant literature in order to determine age-specific transition rates at each stage. The progression and regression rates were converted to annual transition probabilities using the method of Miller and Homan (1994), and these are given in Table 1

**Table 1:** Annual transition probabilities for the natural history model

**Parameter**	**Annual transition probability**		**References**
Uninfected to Cervical HPV infection (HPV incidence)	Age		
	16–19	0.0855	Barnabas and Garnett, 2004; Schiffman and Kjaer, 2003; Melkert *et al*, 1993
	20–24	0.2500	
	25–29	0.1500	
	30–34	0.0576	
	35–39	0.0333	
	40–44	0.0333	
	45–49	0.0333	
	50+	0.0222	
			
Cervical HPV infection to Uninfected	Age		
	16–29	0.7000	Myers *et al*, 2000; Hildesheim *et al*, 1994; Moscicki *et al*, 1998; Koutsky *et al*, 1992; Ho *et al*, 1998; Molano *et al*, 2003
	30+	0.4130	
			
Cervical HPV infection to CIN 1 or CIN 2	0.0959		Myers *et al*, 2000; Moscicki *et al*, 1998
Proportion of Cervical HPV infection progressing directly to CIN 2	0.1350		Myers *et al*, 2000; Schlecht *et al*, 2001
			
CIN 1 to Cervical HPV infection or Uninfected	Age		
	16–34	0.2248	Myers *et al*, 2000; Yokoyama *et al*, 2003; Syrjanen *et al*, 1992; de Brux *et al*, 1983
	35+	0.1124	
			
Proportion CIN 1 regressing directly to Uninfected	0.90		Syrjanen *et al*, 1992
			
CIN 1 to CIN 2	Age		
	16–34	0.0297	de Brux *et al*, 1983; Syrjanen *et al*, 1992; Myers *et al*, 2000
	35+	0.1485	
			
CIN 1 to CIN 3	0.0301		Yokoyama *et al*, 2003
			
CIN 2 to Cervical HPV infection or Uninfected	0.1901		Yokoyama *et al*, 2003
Proportion CIN 2 regressing directly to Uninfected	0.90		Syrjanen *et al*, 1992
			
CIN 2 to CIN 1	0.2430		Syrjanen *et al*, 1992
			
CIN2 to CIN 3	Age		
	16–34	0.0389	Syrjanen *et al*, 1992; de Brux *et al*, 1983; Yokoyama *et al*, 2003
	35–44	0.0797	
	45+	0.1062	
			
CIN 3 to Cervical HPV infection or Uninfected	Age		
	16–44	0.0135	Syrjanen *et al*, 1992
	45+	0.0100	
Proportion CIN 3 regressing directly to Uninfected	0.50		Syrjanen *et al*, 1992
			
CIN 3 to CIN 1	0		—
CIN 3 to CIN 2	0.0135		—
			
CIN 3 to Invasive cervical cancer	0.0099		Syrjanen *et al*, 1992; Ostor, 1993; McIndoe *et al*, 1984
			
Invasive cervical cancer to Death from invasive cervical cancer	0.025		—
			
Transitions to Death from other cause	Age		
	16–24	0.0003	Office of National Statistics, 2003a
	25–34	0.0004	
	35–44	0.0010	
	45–54	0.0026	
	55–64	0.0066	
	65–74	0.0192	

. As young women more commonly have transient infections than older women (Hildesheim *et al*, 1994), age-dependent transition rates from asymptomatic HPV infection to CIN and age-dependent transitions between the various grades of CIN were incorporated into the model.

### Screening and treatment model component

We chose model parameters to simulate screening and treatment according to national guidelines for the NHSCSP and with the reported efficacy of the NHSCSP (Department of Health, 2001). In accordance with pre-2003 UK practice, women aged between 20 and 64 years have been invited for cytological smear screening, with follow-up, referral for evaluation and/or treatment being dependent upon the screening result. The model incorporates the reported age-specific distribution of 5-year screening coverage rates in England, with over 80% of women between 25 and 64 years screened every 5 years (see Table 2

**Table 2:** Parameters for screening and treatment model

**Screening parameters**	**Baseline parameter value**		**Scenario analysis range**	**Reference for baseline parameter value**
Screening coverage	Age	Coverage	Extend screening to women aged 65–79 years	Department of Health, 2001
	25–29	77.0%		
	30–34	83.0%		
	35–39	85.0%	Increase coverage to 77% in women aged 20–24	
	40–44	85.5%		
	45–49	86.0%		
	50–55	85.0%		
	55–59	82.5%		
	60–64	77.5%		
	(average rate 83% over 5 years)			
				
Screening interval	3–5 years		2003 recommendations: no screening ages 20–24; 3 years ages 25–49; 5 years ages 50–64	Department of Health, 2001; Sasieni *et al*, 2003
				
Pap sensitivity	55.6%		40–90%	Nanda *et al*, 2000
				
Proportion HPV-positive post treatment	27%		—	Bodner *et al*, 2002
				
Treatment success (failure due to noncompliance or misdiagnosis)	90%		—	—

).

The assumed sensitivity of the Papanicolaou smear for CIN 2/3 and cancer lesions in the baseline model was derived from a meta-analysis (Nanda *et al*, 2000). An adjustment was applied in order to account for the slightly higher effective cytology threshold required for colposcopy referral in the UK, since women with a single equivocal (borderline changes) or low-grade (mild dyskaryosis) smear result are not automatically referred. We assumed that the imminent transition to liquid-based cytology in the UK will significantly reduce the rate of unsatisfactory smears, but will have a limited effect on the sensitivity of detection of high-grade disease (National Institute for Clinical Excellence, 2003). Therefore, our baseline model is equally valid for conventional cytological screening or in the context of the imminent introduction of liquid-based cytology to the screening programme in the UK. The sensitivity of screening can be varied in the model as appropriate, to reflect the sensitivity of technologies such as HPV DNA testing, or adjunctive test combinations.

We assumed that all unsatisfactory smears were repeated and that follow-up of borderline and mild dyskaryosis smears and any subsequent referrals to colposcopy were completed within 1 year. In accordance with current UK practice, we assumed that confirmed CIN 1 lesions are monitored colposcopically rather than immediately treated. Once referred to colposcopy, we assumed that 90% of women with a CIN 2, CIN 3 or invasive cancer received appropriate follow-up and treatment within the year that they were referred. The remaining 10% of women were assumed to remain untreated due either to colposcopic misclassification of disease or patient noncompliance. For treated women, we assumed eradication of cervical HPV infection in 73% of cases (Bodner *et al*, 2002) and in the remaining 27% we assumed eradication of the CIN lesion, but persistence of cervical HPV infection.

### Model outputs

The model output was the annual age-specific incidence of invasive cervical cancer. This provides a prediction of the number of women with cervical cancer at each age, per 100 000 women of the same age, by single years of age from 16 to 79. We then calculated several summary measures of the predicted incidence, as follows: (1) the age-specific incidence averaged across 5-year age bands and presented graphically, which allows direct comparison with age-specific incidence rates in England; (2) the cumulative lifetime incidence of invasive cervical cancer, from age 20 to 80 years, expressed as a percentage; and (3) the age-standardised incidence rate, standardised to the European standard population (Esteve *et al*, 1994). To permit direct comparisons of the model predictions of incidence rates with England cancer registration rates, we did not account for women who had a hysterectomy for reasons unrelated to cervical neoplastic disease. Not adjusting for hysterectomy allows direct comparison with national cervical cancer incidence rates (Redburn and Murphy, 2001), but does not affect the comparison between the outcomes arising from various interventions.

### Sensitivity and scenario analysis

Sensitivity analysis was used to assess the effect of varying individual model parameters during the construction and testing of the model. Following validation of the baseline model output against observed cancer incidence rates in the UK, scenario analysis was used to explore the impact of changes to the screening test sensitivity, the age of women invited for screening and the screening interval in different age groups.

### Software

Markov cohort analysis was performed using DATA 4.0.7 Software (TreeAge Software, Williamstown, MA, USA). Post-processing of the data was performed using STATA 7.0 (Stata Corp, College Station, TX, USA).

## RESULTS

### HPV prevalence

**Figure 2:**
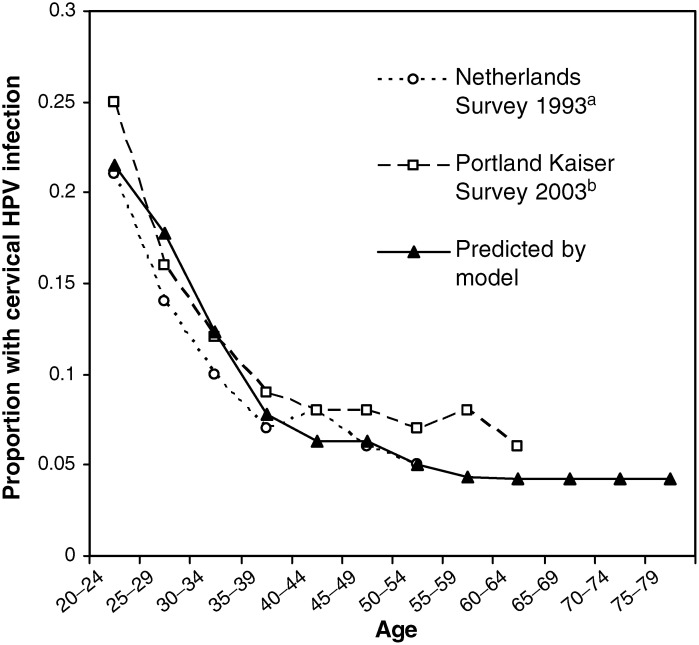
Prevalence of cervical HPV infection (PCR detectable HPV DNA) found in population surveys in the Netherlands and the USA, compared to the model prediction. ^a^Melkert *et al* (1993); ^b^Schiffman and Kjaer (2003).

The model prediction of the age-specific prevalence of HPV infection in women without CIN is given in Figure 2

. The model was calibrated against two sources of data for cross-sectional age-specific prevalence of HPV DNA positivity, based on PCR testing. A survey in the Netherlands (Melkert *et al*, 1993) assessed cross-sectional prevalence of all HPV types in approximately 1700 cytologically normal women. The Portland Kaiser survey in the USA involved an assessment of the prevalence of oncogenic HPV types in more than 20 000 women (Schiffman and Kjaer, 2003). Survey data on HPV prevalence in women aged over 65 years were not available for comparison with the model results. At ages 20–64, satisfactory agreement between the model-predicted and observed prevalence is observed, with a peak HPV prevalence of 20–25% at age 20–24 years, declining thereafter to less than 10% in women over 35 years of age.

### Invasive cancer incidence

**Figure 3:**
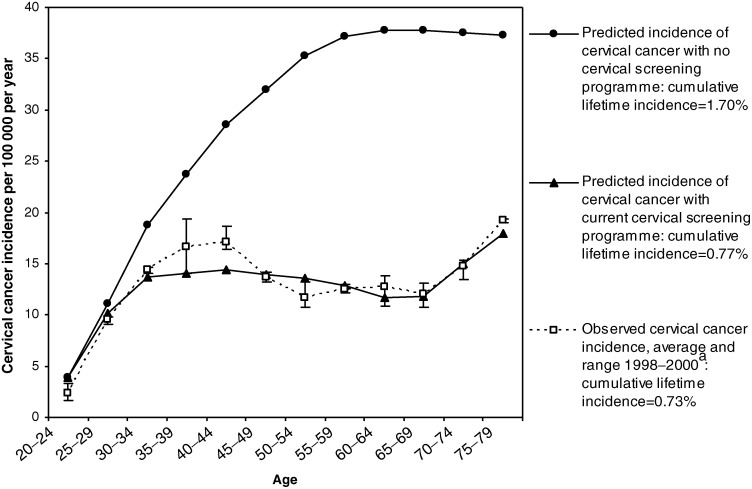
Observed and predicted incidence of invasive cervical cancer in the UK. ^a^Cancer Registrations in England, 1998–2000. Office of National Statistics.

The predicted age-specific annual incidence of invasive cervical cancer in the UK population in the absence of screening is given in Figure 3

. Assuming no screening, the model predicts a cumulative lifetime incidence of cervical cancer of 1.70% and an age-standardised incidence of 18.6 per 100 000 annually. The predicted cancer incidence increases with age until about 55 years and then remains relatively flat, which is in keeping with observed trends in unscreened populations (Beral *et al*, 1994). Figure 3 also shows the predicted effect of the pre-2003 UK screening programme on the incidence of invasive cancer, which agrees closely with England cancer registration data for annual cervical cancer incidence rates (Office of National Statistics, 2001, 2003b). Average age-specific cervical cancer incidence rates for the years 1998–2000 are shown, with bars indicating the range of values observed over the 3 years. The predicted age-specific incidence curve is within the observed 3-year range at most ages, with the possible exception of ages 40–44, where the model predicts slightly lower than observed incidence rates. Overall, the model predicts a reduction in the cumulative lifetime incidence to 0.77% with pre-2003 screening patterns, which is close to the estimated lifetime incidence of 0.73% based on 1998–2000 UK age-specific incidence rates (Office of National Statistics, 2001, 2003b). The model-predicted age-standardised incidence is 8.7 per 100 000 women per year, which is also close to the average cancer registration rate of 9.0 per 100 000 observed from 1998–2000 in the UK (Office of National Statistics, 2001, 2003b). The cancer registration data show an increase in age-specific incidence rates for women aged over the age of 65 years, following the cessation of screening, and this pattern is also observed in the model results.

### Effect of changes to screening practice

Figure 4

**Figure 4:**
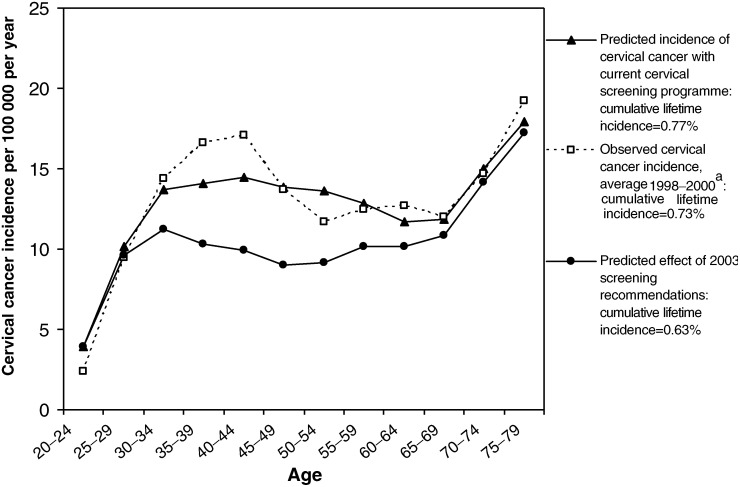
Predicted effect of 2003 NHS Cervical Screening Programme recommendations. ^a^Cancer Registrations in England, 1998–2000. Office of National Statistics.

shows the predicted effect of implementing the 2003 NHSCSP recommended changes in screening intervals, assuming that current levels of population coverage are maintained with more frequent screening in younger women. The age-specific cancer incidence curve demonstrates that the additional benefits of 3-yearly screening in women aged under 50 years would still be evident when these women reached the age 50–69 years, due to increased early detection and treatment of precancerous lesions. The model predicts that the changes in screening intervals will further reduce the cumulative lifetime incidence of cervical cancer by 18% compared to pre-2003 screening, from 0.77 to 0.63%. Compared to no screening at all in the UK population, which corresponds to an estimated lifetime incidence of 1.70% (Figure 3), the overall proportion of cancers prevented in women aged 20–79 years after implementing the 2003 NHSCSP recommendations is estimated to be 63%, with a predicted age-standardised incidence of 6.9 per 100 000 women.

Figure 5

**Figure 5:**
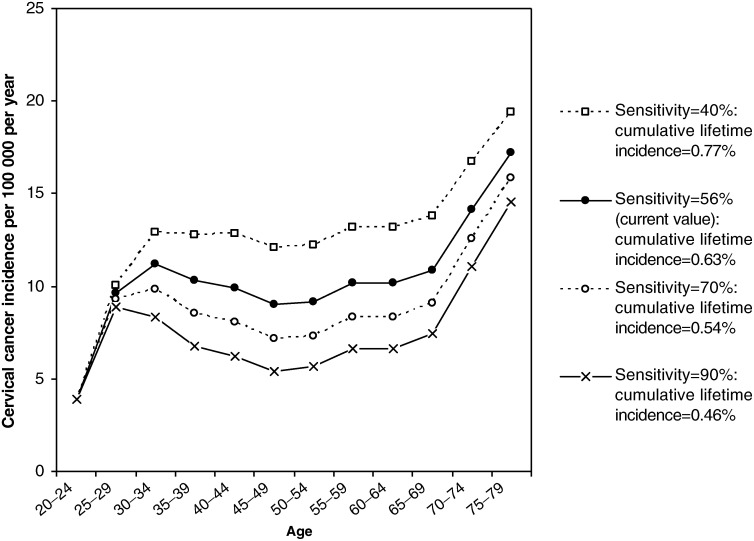
Effect of varying the sensitivity of cervical screening, after implementing the 2003 screening interval changes.

shows the effect of incorporating the 2003 screening interval recommendations and then varying the sensitivity of the screening test (or combination of tests) between 40 and 90%, assuming that the average sensitivity of the cytological smear is 56% (Nanda *et al*, 2000). Increasing the screening sensitivity to 70% would result in an estimated further reduction of cumulative lifetime incidence of 14% from 0.63 to 0.54%, and increasing the sensitivity to 90% or higher would result in a further lifetime incidence reduction of 27%, to 0.46%. Such improvements in screening sensitivity could potentially be achieved, for example, with primary HPV DNA testing (Cuzick *et al*, 2003). In contrast, a 22% increase in the lifetime incidence of cervical cancer (to 0.77%) is predicted if the screening test sensitivity is decreased to 40%.

Figure 6

**Figure 6:**
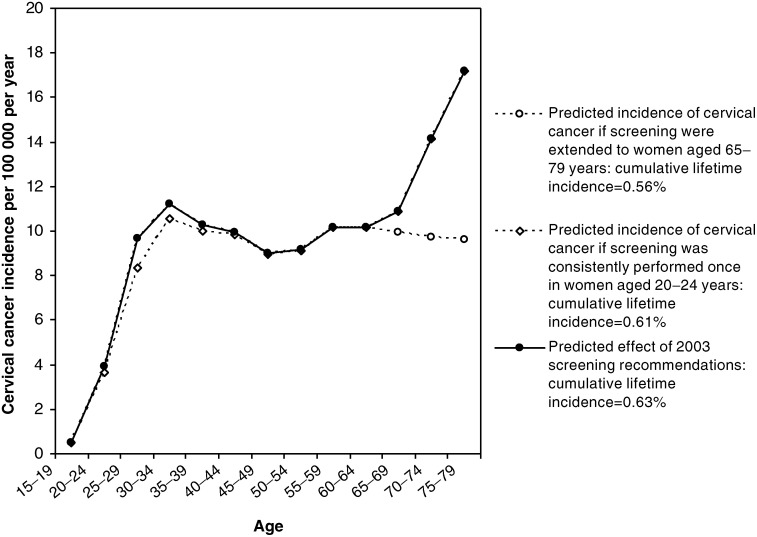
Effect of extending screening to women aged 20–24 or 65–79 years, after implementing the 2003 screening interval changes.

shows the effect of incorporating the 2003 screening interval recommendations and then varying the age of women invited for screening. Increasing the population coverage age range by extending screening to women aged 65–79 years would result in a predicted further decrease of 11% in cumulative lifetime incidence, from 0.63 to 0.56%. However, continuing to systematically invite women in the 20–24 year age group once in the 5-year period would result in a minimal 3% decrease in the lifetime incidence of cervical cancer to 0.61%, even if coverage rates of over 75% were achieved in this age group.

## DISCUSSION

Although the molecular events involved in cervical HPV infection and the development of neoplasia are not completely understood, the clinical course of cervical carcinogenesis is now relatively well charted. Therefore, the natural history of cervical disease lends itself to a modelling approach. Modelling can assist with decision making by exploring the implications of a range of possible interventions (Royston, 1999) and has been previously used to evaluate the natural history of CIN (Myers *et al*, 2000) and changes in screening practice (Sherlaw-Johnson *et al*, 1994, 1999). The model we have described builds on previous work to provide a tool that encompasses the current understanding of the natural history of cervical neoplasia and is specific to the UK setting.

We used the Markov simulation of the events leading to cervical cancer and the screening and treatment of cervical disease to predict cancer incidence rates in the UK population under various conditions. The predicted age-specific cervical cancer incidence in the absence of screening was 18.6 per 100 000 women, standardised to the European standard population. The cancer registration data, standardised to the same population, suggest rates between 14 and 16 per 100 000 during the 1970s and 1980s in England (Quinn *et al*, 1999). Some opportunistic cervical screening was performed in the UK from the 1960s and, as expected, the observed incidence in the context of opportunistic screening was slightly less than the model prediction for a completely unscreened population. Since cancer registration data for England, which has only been collected since the early 1970s, incorporates a screening effect, they cannot be used to validate the model prediction for the incidence of cervical cancer in the UK population in the absence of screening. There are additional problems associated with using historical data, in terms of disentangling the influence of cohort effects related to differential HPV exposure and the influence of changing practice in screening, diagnostic and treatment procedures. For these reasons, we thought it most appropriate to estimate the validity of the model by incorporating the effect of pre-2003 screening practice and comparing the model predictions of cervical cancer incidence rates with recent cancer registration data for the UK. We found close agreement between the observed and predicted values of age-specific cervical cancer incidence, cumulative lifetime incidence and age-standardised incidence in the screened UK population (Figure 3).

The primary objective of the study was to assess the likely long-term impact of the 2003 NHSCSP recommended changes in screening intervals. Although the current study has focused upon screening in the UK, a Working Group of the International Agency for Research on Cancer (IARC) has recently confirmed the efficacy of screening every 3 years between the ages of 25 and 49 years and every 5 years between the ages of 50 and 64 years (International Agency for Research on Cancer, 2004), and therefore our results also have more general implications. We found that ceasing screening in women under the age of 25 years will have a very small effect on the lifetime incidence of cervical cancer. Reducing the screening interval to 3 years in women aged 25–49 will have major benefits, resulting in an 18% reduction of the cumulative lifetime incidence of cervical cancer, compared to pre-2003 screening. As a result, the predicted proportion of cervical cancers prevented in women aged 20–79, compared to an unscreened population, is 63%. Our findings of a reduction in cervical cancer incidence broadly agree with the original estimates performed by Sasieni *et al* (2003), who found that the new screening intervals would be associated with a 61% reduction in women aged 20–39 years and an 84% reduction in women aged 40–54 years, compared to women who were never screened. However, the results are not directly comparable because our model included microinvasive cancers, whereas their estimate was based on 1305 women aged 20–69 years with Stage 1B+ cancer and 2532 age-matched control women, but did not include 537 microinvasive (Stage 1A) cancers and 490 cancers of unknown stage observed in women of the same age group. Also, Sasieni *et al* assumed that all women with abnormal smears are prevented from developing cancer, which is not assumed in our model. They calculated the relative risks resulting from 3- or 5-year screening intervals in various age groups as a function of the time since the last negative smear, and then derived a summary measure of the overall proportion of cancers preventable, using a previously described method (Sasieni *et al*, 1996). This approach provided a measure of the efficacy of screening in individuals who complied with screening recommendations. Our study extends these results by assessing the overall effectiveness of the proposed NHSCSP changes across the lifetime of a cohort of women, taking into account real-world limitations in population screening coverage rates, the sensitivity of screening and the efficacy of treatment. For women over 50 years of age, the modelling approach takes into account the carried-over beneficial effect resulting from previous 3-yearly screening of the cohort when they were aged 25–49 years, whereas this carry-over effect could not be taken into account in the relative risk estimates performed by Sasieni *et al*.

Some further reductions in cervical cancer incidence could be achieved after the implementation of the 2003 screening interval recommendations, by means of increasing the sensitivity of the screening test or extending screening to older women. Increasing the sensitivity of the screening test would result in a substantial further reduction of the cumulative lifetime incidence of cervical cancer, of up to 27%. Alternatively, when we examined the possibility of extending screening to include 5-yearly coverage of women aged 65–79 years, we found that there was an 11% further reduction in cumulative lifetime incidence predicted. However, this measure does not take into account the person-years saved, and reducing incidence in women over 65 years of age is expected to save fewer years of life compared to reducing incidence in younger women. Therefore, when comparing the two possible strategies, both the relative magnitudes of the expected improvements and the age ranges over which incidence is reduced would suggest that increasing screening sensitivity would be the most preferable option for the future. However, cost-effectiveness analysis is required to fully assess the public health impact of such future screening strategies.

In conclusion, a UK-specific Markov model for cervical HPV infection, CIN and invasive cervical cancer has been constructed, successfully validated against cancer registration data and used as a tool to evaluate the impact of screening policy changes. The results support the 2003 NHSCSP recommendations that screening should start at age 25 years, that screening intervals should be reduced to 3 years in women aged 25–49 years and that screening intervals should be standardised to 5 years in those aged 50–64, and suggest that a substantial further reduction in cervical cancer incidence should follow.

## References

[bib1] Barnabas RV, Garnett GP (2004) The potential public health impact of vaccines against human papillomavirus. European Consortium for Cervical Cancer Education, (in press)

[bib2] Beral V, Hermon C, Munoz N, Devesa SS (1994) Cervical cancer. Cancer Surv 19–20:265–285 7534630

[bib3] Bodner K, Bodner-Adler B, Wierrani F, Kimberger O, Denk C, Grunberger W (2002) Is therapeutic conization sufficient to eliminate a high-risk HPV infection of the uterine cervix? A clinicopathological analysis. Anticancer Res 22:3733–3736 12552985

[bib4] Cuzick J, Szarewski A, Cubie H, Hulman G, Kitchener H, Luesley D, McGoogan E, Menon U, Terry G, Edwards R, Brooks C, Desai M, Gie C, Ho L, Jacobs I, Pickles C, Sasieni P (2003) Management of women who test positive for high-risk types of human papillomavirus: the HART study. Lancet 362:1871–1876 14667741 10.1016/S0140-6736(03)14955-0

[bib5] de Brux J, Orth G, Croissant O, Cochard B, Ionesco M (1983) Condylomatous lesions of the uterine cervix: their course in 2466 patients. Bull Cancer 70:410–422 6686786

[bib6] Department of Health (2001) Department of Health Bulletin 2001/22, Cervical Screening Programme, England, 2000–2001, September 2001

[bib7] Esteve J, Benhamou E, Raymond L (1994) Descriptive Epidemiology, IARC Scientific Publications No. 128. IV. Lyon, France: International Agency for Cancer Research 7698823

[bib8] Hildesheim A, Schiffman MH, Gravitt PE, Glass AG, Greer CE, Zhang T, Scott DR, Rush BB, Lawler P, Sherman ME (1994) Persistence of type-specific human papillomavirus infection among cytologically normal women. J Infect Dis 169:235–240 8106758 10.1093/infdis/169.2.235

[bib9] Ho GY, Bierman R, Beardsley L, Chang CJ, Burk RD (1998) Natural history of cervicovaginal papillomavirus infection in young women. N Engl J Med 338:423–428 9459645 10.1056/NEJM199802123380703

[bib10] International Agency for Research on Cancer (IARC) (2004) www.iarc.fr/pageroot/PRELEASES/pr151a.html

[bib11] Koutsky LA, Holmes KK, Critchlow CW, Stevens CE, Paavonen J, Beckmann AM, DeRouen TA, Galloway DA, Vernon D, Kiviat NB (1992) A cohort study of the risk of cervical intraepithelial neoplasia grade 2 or 3 in relation to papillomavirus infection. N Engl J Med 327:1272–1278 1328880 10.1056/NEJM199210293271804

[bib12] McIndoe WA, McLean MR, Jones RW, Mullins PR (1984) The invasive potential of carcinoma *in situ* of the cervix. Obstet Gynecol 64:451–458 6483293

[bib13] Melkert PW, Hopman E, Van Den Brule AJ, Risse EK, van Diest PJ, Bleker OP, Helmerhorst T, Schipper ME, Meijer CJ, Walboomers JM (1993) Prevalence of HPV in cytomorphologically normal cervical smears, as determined by the polymerase chain reaction, is age-dependent. Int J Cancer 53:919–923 8386137 10.1002/ijc.2910530609

[bib14] Miller DK, Homan SM (1994) Determining transition probabilities: confusion and suggestions. Med Decis Making 14:52–58 8152357 10.1177/0272989X9401400107

[bib15] Molano M, Van den Brule A, Plummer M, Weiderpass E, Posso H, Arslan A, Meijer CJ, Munoz N, Franceschi S (2003) Determinants of clearance of human papillomavirus infections in Colombian women with normal cytology: a population-based, 5-year follow-up study. Am J Epidemiol 158:486–494 12936904 10.1093/aje/kwg171

[bib16] Moscicki AB, Shiboski S, Broering J, Powell K, Clayton L, Jay N, Darragh TM, Brescia R, Kanowitz S, Miller SB, Stone J, Hanson E, Palefsky J (1998) The natural history of human papillomavirus infection as measured by repeated DNA testing in adolescent and young women. J Pediatr 132:277–284 9506641 10.1016/s0022-3476(98)70445-7

[bib17] Myers ER, McCrory DC, Nanda K, Bastian L, Matchar DB (2000) Mathematical model for the natural history of human papillomavirus infection and cervical carcinogenesis. Am J Epidemiol 151:1158–1171 10905528 10.1093/oxfordjournals.aje.a010166

[bib18] Nanda K, McCrory DC, Myers ER, Bastian LA, Hasselblad V, Hickey JD, Matchar DB (2000) Accuracy of the Papanicolaou test in screening for and follow-up of cervical cytologic abnormalities: a systematic review. Ann Intern Med 132:810–819 10819705 10.7326/0003-4819-132-10-200005160-00009

[bib19] National Institute for Clinical Excellence (2003) Final Appraisal Determination – Guidance on the Use of Liquid-Based Cytology for Cervical Screening. Review of Existing Guidance Number 5

[bib20] Office of National Statistics (2001) Cancer Statistics: Registrations., No. 28, England, 1995–1997. London, UK: Office of National Statistics

[bib21] Office of National Statistics (2003a) Mortality Statistics: Cause. Review of the Registrar General on Deaths by Cause, Sex and Age, In England and Wales, 1999, No. 26. London, UK: Office of National Statistics

[bib22] Office of National Statistics (2003b) National Statistics: Cancer Registrations in England, 2000. London, UK: Office of National Statistics

[bib23] Ostor AG (1993) Natural history of cervical intraepithelial neoplasia: a critical review. Int J Gynecol Pathol 12:186–192 8463044

[bib24] Quinn M, Babb P, Jones J, Allen E (1999) Effect of screening on incidence of and mortality from cancer of cervix in England: evaluation based on routinely collected statistics. BMJ 318:904–908 10102852 10.1136/bmj.318.7188.904 PMC27810

[bib25] Redburn JC, Murphy MF (2001) Hysterectomy prevalence and adjusted cervical and uterine cancer rates in England and Wales. Br J Obstet Gynaecol 108:388–395 10.1111/j.1471-0528.2001.00098.x 11305546

[bib26] Royston G (1999) Commentary: trials *vs* models in appraising screening programmes. BMJ 318:360–361 10075469

[bib27] Sasieni P, Adams J, Cuzick J (2003) Benefit of cervical screening at different ages: evidence from the UK audit of screening histories. Br J Cancer 89:88–93 12838306 10.1038/sj.bjc.6600974 PMC2394236

[bib28] Sasieni PD, Cuzick J, Lynch-Farmery E (1996) Estimating the efficacy of screening by auditing smear histories of women with and without cervical cancer. The National Co-ordinating Network for Cervical Screening Working Group. Br J Cancer 73:1001–1005 8611418 10.1038/bjc.1996.196 PMC2075813

[bib29] Schiffman M, Kjaer SK (2003) Chapter 2: Natural history of anogenital human papillomavirus infection and neoplasia. J Natl Cancer Inst Monogr 31:14–19 10.1093/oxfordjournals.jncimonographs.a003476 12807940

[bib30] Schlecht NF, Kulaga S, Robitaille J, Ferreira S, Santos M, Miyamura RA, Duarte-Franco E, Rohan TE, Ferenczy A, Villa LL, Franco EL (2001) Persistent human papillomavirus infection as a predictor of cervical intraepithelial neoplasia. JAMA 286:3106–3114 11754676 10.1001/jama.286.24.3106

[bib32] Sherlaw-Johnson C, Gallivan S, Jenkins D, Jones MH (1994) Cytological screening and management of abnormalities in prevention of cervical cancer: an overview with stochastic modelling. J Clin Pathol 47:430–435 8027396 10.1136/jcp.47.5.430 PMC502020

[bib31] Sherlaw-Johnson C, Gallivan S, Jenkins D (1999) Withdrawing low risk women from cervical screening programmes: mathematical modelling study. BMJ 318:356–360 9933195 10.1136/bmj.318.7180.356 PMC27721

[bib33] Syrjanen K, Kataja V, Yliskoski M, Chang F, Syrjanen S, Saarikoski S (1992) Natural history of cervical human papillomavirus lesions does not substantiate the biologic relevance of the Bethesda System. Obstet Gynecol 79:675–682 1314359

[bib34] Yokoyama M, Iwasaka T, Nagata C, Nozawa S, Sekiya S, Hirai Y, Kanazawa K, Sato S, Hoshiai H, Sugase M, Kawana T, Yoshikawa H (2003) Prognostic factors associated with the clinical outcome of cervical intraepithelial neoplasia: a cohort study in Japan. Cancer Lett 192:171–179 12668281 10.1016/s0304-3835(02)00715-2

